# Spontaneous Breathing Rate Variations Linked to Social Exclusion and Emotion Self-assessment

**DOI:** 10.1007/s10484-022-09551-5

**Published:** 2022-06-13

**Authors:** Antonio R. Hidalgo-Muñoz, Esther Cuadrado, Rosario Castillo-Mayén, Bárbara Luque, Carmen Tabernero

**Affiliations:** 1 Instituto de Neurosciencias de Castilla y León (INCYL), University of Salamanca, Salamanca, Spain; 2 Department of Psychology, University of Cordoba, Córdoba, Spain; 3 Maimonides Biomedical Research Institute of Cordoba (IMIBIC), Córdoba, Spain

**Keywords:** Affective state, Breathing rate, Emotion, Metaemotion, Social exclusion

## Abstract

The emotional reactions to social exclusion can be associated with physiological responses that could allow researchers to estimate the valence and intensity of the ongoing affective state. In this work, respiratory activity was analysed to verify whether breathing rate variations can be considered as predictive factors of subsequent positive and negative affect after inclusion and exclusion in young women. A standard Cyberball task was implemented and manipulated information was provided to the participants to create both conditions. The participants were socially excluded by limiting their participation to 6% of the total number of passes among three teammates and providing negative feedback about them. The results suggest that breathing rate can be a good option to infer subjective feelings during social interactions and a promising feature to incorporate into modern emotion monitoring systems as an alternative to other physiological measures. Furthermore, the interaction between metaemotion and physiology was studied by recording breathing rate while completing the Positive and Negative Affect Schedule, evidencing a breathing rate increase during the emotion self-assessment only after exclusion.

Social interaction elicits different types of emotions. In the case of social exclusion or rejection situations, negative emotions such as anger, sadness, guilt or embarrassment usually emerge (Leary, 2015), in addition to arousal increases. These affective reactions are usually accompanied by physiological responses from the autonomic nervous system (Kreibig, 2010). Although these body manifestations can be affected by specific personality traits (Hidalgo-Muñoz et al., 2021; Stemmler & Wacker, 2010), some physiological patterns seem to be similar across people coping with difficult social interactions, even when different levels of fear to negative social evaluations were present (Chen & Drummond, 2008). Therefore, it would be possible to associate physiological response modulations with emotion dynamics in specific interpersonal contexts.

Arguably, cardiovascular activity is one of the most commonly used measures to analyse psychophysiological responses and to discriminate between high arousal emotions linked to social interactions. For instance, Liddell and Courtney (2018) have analysed heart rate (HR) and HR variability (HRV) to examine physiological reactions in the case of social exclusion. In this situation, the authors concluded that attachment style has an influence on the cardiovagal balance response. Beames et al. (2019), in their review, compile numerous features extracted from cardiac activity to study the physiological correlates of anger and aggressive behaviours linked to social interactions. Together with cardiac activity, skin conductance (SC) is another relevant measure to determine emotional reactions. In particular, SC has been used to detect acute stress responses in different situations (Boucsein, 2012), including diverse social situations such as rejection (Iffland et al., 2014) or competition (Binboga et al., 2012) among others. Although HR and SC sensors have been widely developed and implemented in wearable devices, thus allowing the easy acquisition of proper signals, there is another physiological signal that is often overlooked in several research fields despite its potential applications: the respiratory signal (Cretikos et al., 2008). Hence, current and future research on respiration activity and emotions linked to social situations, e.g., embarrassment, is encouraged by researchers (Kreibig, 2010).

The incorporation of respiration signals to study emotions in social contexts shows several advantages. Firstly, respiratory activity recordings can complement other techniques to disentangle the cognitive and the emotional sources modulating the autonomic nervous system responses (Kreibig, 2010), as well as be used to correct features from other recorded signals such as electrocardiograms (Grossman & Taylor, 2007). Secondly, the acquisition and signal processing devices, specifically for breathing rate (BR), are cost-effective and, recently, non-contact, facilitating the monitoring of numerous individuals (Prince et al., 2020), and, specifically, people for whom it is inconvenient to use wearable SC or HR sensors. Indeed, in contrast to most psychophysiological manifestations, BR can be measured by using video or radars (Nejadgholi et al., 2019). Thirdly, respiration can be modulated conscientiously to reduce BR in order to calm stress responses (Arch & Craske, 2006; Zaccaro et al., 2018; Zepf et al., 2021). Thus, modern technologies aiming to regulate emotions by means of response modulation (Gross, 2014) show increasing interest in studying BR in virtual (Blum et al., 2020) or ecological settings (Hidalgo-Muñoz et al., 2019), and even using it to express emotions in virtual reality (de Melo et al., 2010).

Besides the aforementioned practical advantages, respiratory activity is an interesting measure in psychology research. Different respiratory parameters have already been used to discriminate between basic emotions such as anger, happiness or sadness elicited by films (Hameed et al., 2019), using particular experimental tests, such as the cold pressor test (Boiten, 1998) or to estimate cognitive loads (Grassmann et al., 2016). It has also been proposed that the respiratory sinus arrhythmia be included in biofeedback applications to overcome several psychological troubles, e.g., for post-traumatic stress disorder (Lipschutz et al., 2017; Zucker et al., 2009). In the present case of BR, its increase has been associated with high arousal and sustained attention (Vlemincx et al., 2011). BR is considered as an indicator of the stress response, based on the working mechanisms of the sympathetic and parasympathetic autonomic nervous system (Grossman & Taylor, 2007). These findings are in agreement with the BR increases linked to the intensity increase of emotions, such as anger (Rainville et al., 2006). Hence, BR is a respiratory parameter that, although simple, can provide valuable insights into different stages of emotional processing (Philippot et al., 2002).

After verifying the affective state variations due to social inclusion and exclusion situations generated by the Cyberball task, the goal of the present study is twofold. Firstly, to determine the suitability of the spontaneous BR modulations to predict the positive and negative affects after inclusion and exclusion, respectively. Overall, our hypothesis is that the increase of emotions with negative valence due to social exclusion will be associated with BR increases. Secondly, to examine the impact of metaemotion processes (Mitchell, 2020) on the aforementioned BR modulations. This analysis will permit the acquisition of nuance conclusions on the previous question. Metaemotion can allude to the evaluation of the actual emotions, including the differentiation among others and the capacity to label them. In this way, emotions are considered to be the object of the appraisal process. Besides the previous meaning, metaemotion can also refer to the “affective reactions toward the primary emotion, and motivation to change the expected course of the primary emotion” (Bartsch et al., 2008, p. 16). Our hypothesis is that the explicit assessment of emotions will differently modulate or exacerbate the physiological responses according to the actual affective state (Hauser et al., 2018), mainly under high arousal or negative valence emotions (Kassam & Mendes, 2013).

## Materials and Methods

### Participants

Fifty young women (18–22 years) carried out the experiment. They were all healthy without a history of severe medical treatment, with neither cardiorespiratory nor psychological troubles. Two of them had to be withdrawn due to system failures, giving a total *N* = 48 (mean: 19.6 years). The study was approved by the Research Ethics Committee in accordance with the Declaration of Helsinki. Informed consent was obtained by all the volunteers, who were paid 16 euros for their participation.

### Procedure

The procedure was the same as that explained in Cuadrado et al. (2021). The experiment was based on the Cyberball task (4th version), usually employed in studies on exclusion, ostracism, popularity or social anxiety (Williams & Jarvis, 2006).

In the present protocol, firstly, the participants introduced themselves to other virtual participants with a written description (with the possibility of including a photograph). Then, they entered an online platform to play the game and were allowed to read the descriptions of six other participants. They were informed that they were randomly incorporated into a three-players team (with a boy and a girl) and they could, then, start the task. Two equal-size experimental groups were constituted: EXCLUSION and INCLUSION. Participants were randomly allocated to each group. The task was divided into two blocks:

- BLOCK 1 (2.28 min on average) consisted in the Cyberball task, manipulated to create two conditions. In total, the game comprised 30 passes between the three players belonging to the team. For the EXCLUSION group, the participant received the ball twice (6% of the total) from their partners, whereas for the INCLUSION group, the ball was received ten times (33% of the total).
- BLOCK 2 (at least 30 s) started just after the previous block. The participants received manipulated information showing the number of times they had received the ball and the reasons given by their teammates: “a few times because they did not like them” for the EXCLUSION group or “many times because they like them” for the INCLUSION group.

Positive affect (*PA*) and negative affect (*NA*) were assessed twice: before BLOCK 1 and after BLOCK 2 by means of the Positive and Negative Affect Schedule-PANAS (Watson et al., 1988). The original PANAS is a scale composed of 20 items presenting mood-related adjectives such as “ashamed”, “enthusiastic”, “jittery”, etc. Responders are asked to rate the extent to which they experienced these emotions on a 5-point Likert scale ranging from 1 (very slightly or not at all) to 5 (very much). The most common approach to analyse the data from the PANAS, which has been validated in a Spanish population (Díaz-García et al., 2020), is based on a 2-factor structure (*PA* and *NA*) showing good internal consistency (0.91 and 0.87 for *PA* and *NA*, respectively). The time during which they completed the PANAS the first time will be referred as PN1, and the second time as PN2.

### Physiological Signal: Breathing Rate Variations

The respiration signal was recorded by means of the biofeedback package PHYSIOLAB Technologies, J&J Engineering I-330-C2, by using a belt positioned on the chest of the participant. The recording included 5 min of baseline before beginning the task and lasted until the whole experiment was finished. BR values were automatically provided by the software based on respiratory signal peaks. The values for each time interval, i.e., the moments filling the PANAS and during both blocks, were intra-subject baselined as follows:

$$\varDelta {BR}_{interval}=\frac{{BR}_{interval}-{BR}_{baseline}}{{BR}_{baseline}}$$

### Statistical Analysis

Normality was checked using Shapiro–Wilk’s test. T-Student tests were performed if normality could be assumed and Cohen’s *d* computed to estimate size effect. Otherwise, Wilcoxon tests and r parameter for estimating effect size were used. Multiple regression models, without intercept in the equation, were built considering only the significant *ΔBR* variables. Correlation was computed based on Spearman’s *rho*. All the analyses were performed in JASP 0.14, whilst Gpower 3.1 software was used to verify the suitable statistical power considering the actual sample size after data exclusion, achieving power values of at least 0.80 for mean contrast analyses and more than 0.85 for regression models.

For the analysis, the independent variable was the group to which the participants belonged: INCLUSION or EXCLUSION, whereas the dependent variables were the BR variations (Δ*BR*) for four conditions (PN1, BLOCK 1, BLOCK 2, PN2) and the self-reported score of positive (*PA*) and negative affect (*NA*) measured by the PANAS (only measured for PN1 and PN2).

## Results

### Positive and Negative Affect Variations Due to Social Inclusion and Exclusion

In PN1, the affective state did not differ between groups (*p* = .943; *p* = .223 for *PA* and *NA*, respectively), where the values of *PA* were *M* = 5.32, *SD* = 1.03; and the values of *NA* were *M* = 1.68; *SD* = 0.79. In contrast, in PN2, *PA* was higher [*t*(46) = 3.433; *p* < .001; *d* = 0.991] for the INCLUSION (*M* = 5.44; *SD* = 0.95) than for the EXCLUSION group (*M* = 4.22; *SD* = 1.45). The inverse pattern was found for *NA* (*Z* = 3.786; *p* < .001; *r* = − .547), where *NA* was lower for the INCLUSION (*M* = 1.27; *SD* = 0.46) than for the EXCLUSION (*M* = 2.48; *SD* = 1.52) group.

**Fig. 1:**
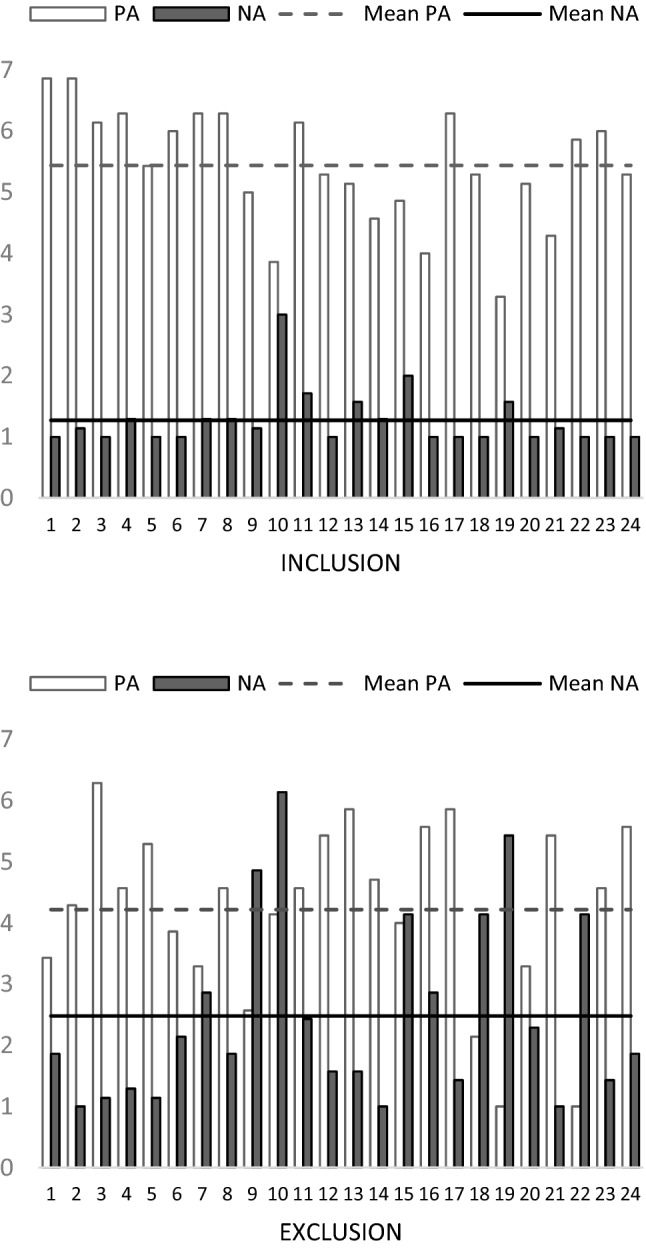
Positive (PA) and negative (NA) affect scores after Cyberball for the participants in the INCLUSION and EXCLUSION groups

A significant decrease in *PA* (*Z* = 4.257; *p* < .001; *r* = .614) and an increase in *NA* (*Z* = 2.608; *p* = .005; *r* = − .376) were found in the EXCLUSION group between PN1 and PN2, whereas a significant increase in PA [*t*(23) = 2.35; *p* = .014; *d* = 0.480] and a decrease in *NA* (*Z* = 3.186; *p* < .001; *r* = .460) were found for INCLUSION. Figure 1 shows the values of *PA* and *NA* for every participant for both the INCLUSION and EXCLUSION groups in PN2.

## Breathing Rate Modulation During Metaemotion (PANAS Completion)

Due to system failure, three participants had to be withdrawn from all the respiratory activity analyses (one from INCLUSION and two from EXCLUSION) and two had to be only withdrawn for BLOCK 2. Additionally, three participants were considered to be outliers (deviation greater than 3*SD from the median value of the group) during BLOCK 2.

The ΔBR values in PN1 (*M* = 0.109; *SD* = 0.211) were significantly higher than zero (Z = 4.074; *p* < .001; *r* = .420), and, therefore, BR during PN1 was higher than during baseline. For this analysis, all the participants from both groups were considered, since they had not passed the experimental condition yet.

The median of the BR, considering all the participants, during baseline was 17.18 bpm (Inter Quartile Range = 3.530).

## Effect of Exclusion and Inclusion on Breathing Rate Variations

**Fig. 2:**
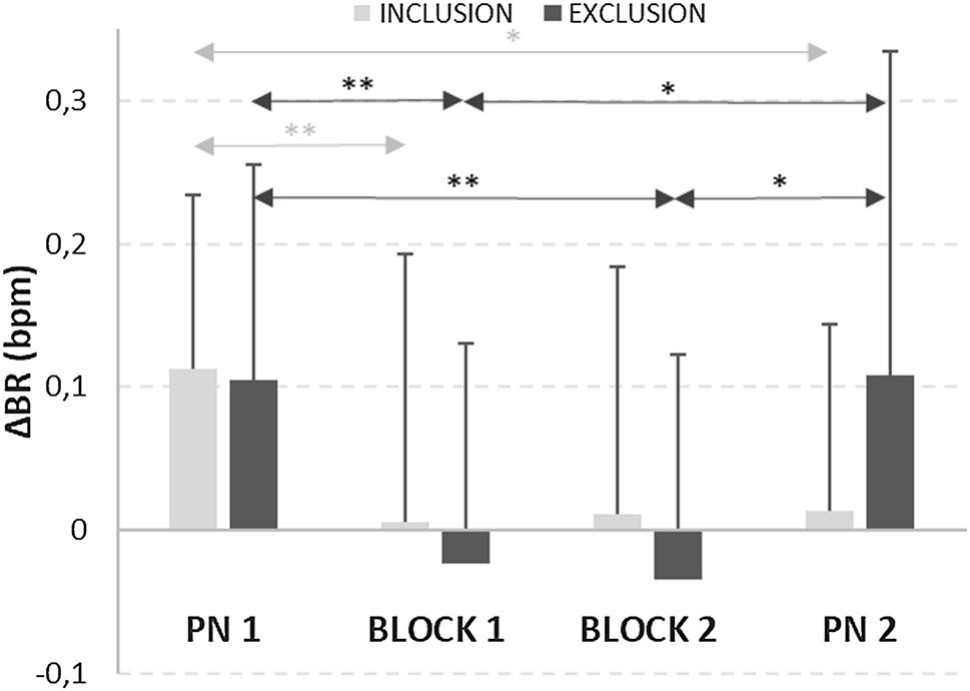
Variations of BR (breaths per minute) compared to basal level for the different time intervals (*p < .05; **p < .01)

Figure 2 depicts ΔBR values and significant differences for all the experimental conditions. A significant ΔBR decrease in BLOCK 1 compared to PN1 was found for both the EXCLUSION [*t*(22) = 3.23; *p* = .002; *d* = 0.674] and INCLUSION groups (*t*(23) = 2.61; *p* = .008; *d* = 0.532).

No significant differences in ΔBR were found in either inter-group during any block nor between BLOCK 1 and BLOCK 2. However, ΔBR was significantly lower in BLOCK 2 compared to PN1 for the EXCLUSION group (*Z* = 2.516; *p* = .006; *r* = .379). Besides, ΔBR was significantly lower in PN2 than PN1 for the INCLUSION group (*Z* = 2.057; *p* = .020; *r* = − .297) and ΔBR was higher in PN2 than both BLOCK 1 (*Z* = 3.224; *p* < .001; *r* = .475) and BLOCK 2 (*Z* = 2.094; *p* = .036; *r* = .316) for the EXCLUSION group.

Regarding the significant correlation values between respiration variations in BLOCK 2 and affect (PN2), a positive correlation was found with *NA* (*rho* = 0.480; *p* = .032) and a negative correlation with *PA* (*rho* = − 0.456; *p* = .043) in PN2 for only the INCLUSION group.

## Prediction of Positive and Negative Affect After Cyberball

Table 1 shows the significant coefficients and associated parameters for the regression models to predict the intensity of the most relevant valence according to emotion reaction (*PA* for INCLUSION group and *NA* for EXCLUSION group, since the variability of the other valence was supposed to be small, as found in the previous results) at the end of the experiment. ΔBR values for every condition were entered as initial inputs to the models and no intercept was considered.

**Table 1:** Regression models for positive (PA) and negative (NA) affects in the INCLUSION and EXCLUSION groups after Cyberball task (PN2)

Dependent variable	*R*	*R*^2^	Predictive variables	Standardized β	*t*	Sig.
***PA***_INCLUSION_	0.502	0.252	ΔBR_PN1_	2.582	2.53	0.020
***NA***_EXCLUSION_	0.564	0.318	ΔBR_BLOCK1_	− 1.924	− 2.70	0.014
			ΔBR_PN2_	2.106	3.05	0.006

*R* Correlation coefficient; *R*^2^ Coefficient of determination to estimate the prediction quality; *Standardized β* Coefficients that compare the strength of the effect of each individual Predictive variable to the Dependent variable; *t* t-test value to assess whether the coefficient is significantly different from zero; *Sig*. *p*-value associated with *t*. The predictive variables used as independent variables are explained in the “Procedure” section

## Discussion

This study analyses the relationship between the affective states elicited by social exclusion and the spontaneous BR modulations linked to different emotional processing stages. In regard to the subjective affective states, in agreement with previous studies (Beames et al., 2019; Cuadrado et al., 2021), social exclusion increased negative affect and decreased positive affect. An inverse pattern was found for the inclusion situation, where lower values and variability were found for negative affect, whereas positive affect predominated. Concerning the respiratory activity, the results of the present study make a step forward towards the literature’s understanding of the autonomic manifestations, not only those linked to social-related emotions but also to the fact of self-assessment and quantifying these emotions.

Indeed, the first self-assessment of affects during the experiment increased BR for all the participants. This increase could be due to the metaemotion process which involves an interoceptive evaluation, where the physiological arousal would be crucial to make the process effective (Keller et al., 2020). This process is, in turn, cognitively demanding and, consequently, also influences BR (Grassmann et al., 2016). The temporal evolution of the affective state and the physiological modulations due to its evaluation can be related to the dynamic nature of emotion and the reappraisal proposed by Scherer (2009). However, we have to be cautious and not to discard possible effects of anticipation or attentional engagement to the task in the particular experimental setting, as some participants could have completed the scale quickly without a deep introspection process.

Just after the completion of the PANAS, during the first block, BR decreased. This result could reflect an emotion regulation strategy (Koole, 2009), albeit an unconscious one, after the metaemotion process. Other indicators based on amplitude values, such as the sigh rate, or BR variability could confirm whether a psychophysiological resetting occurred to ready the participant for the task (Vlemincx et al., 2011).

Unexpectedly, no difference between groups was found during the game in either the first or the second block. We could hypothesize that during the first block of Cyberball, the participants could have been so engaged by the task that they were not yet completely aware of the social interaction quality. However, this explanation may be partially inconsistent with the sociometer theory which suggests that people are sensitive to and continuously monitoring social exclusion cues (Leary & Baumeister, 2000) and with previous studies which demonstrated that social exclusion simulated by Cyberball could elicit physiological responses (Iffland et al., 2014). One possibility is that BR variations as a consequence of self-conscious emotions involving social cues would need longer to be significant, in contrast to resetting due to stress or to the regulation function.

Furthermore, the possible inter-group difference would have been particularly expected when the negative or positive feedback from teammates was known (BLOCK 2). Specifically, in the exclusion situation, arousal was expected to be increased in comparison to the inclusion condition, due to either embarrassment or anger (Kreibig, 2010), which comprise part of the negative affect, since the rejection became explicit. Either way, a difference between groups was not found until participants completed the PANAS for the second time (i.e., PN2), where the participants had to focus on their feelings.

In the inclusion group, the BR decrease during the second completion of the PANAS compared to the first could be linked to the relief (Kreibig, 2010) or a relaxed state after finishing the experiment. Completing the PANAS for the second time would not have had the same impact than the previous time, since emotion evaluation would be easier, based on slight emotion variations from PN1 and, consequently, without requiring a high demanding interoceptive process. In contrast, this self-evaluation process would be more demanding in the group suffering from social exclusion, where affective valence was more negatively impacted. Arguably, this metaemotion process could have made feelings of exclusion, anger and embarrassment stronger than during the realisation of the task, where attention was instead linked to information processing linked to the game. In other words, the explicit recognition of the negative affect would accentuate the autonomic response, as claimed in Kassam and Mendes (2013) and, therefore, BR would be spontaneously increased. However, to determine the inverse relationship, i.e., whether BR variations increase negative affect, and the impact of interoceptive accuracy and sensitivity on the emotions provoked by social exclusion remains an open question (Zamariola et al., 2019).

Concerning the determination of the ongoing affective state, for predicting positive affect after an inclusionary situation, only the BR during the first completion of PANAS (i.e., PN1, the first metaemotion process) was relevant. It is likely that the participants who particularly engaged in the task from the beginning were the most motivated and also the most sensitive to the positive feedback.

In the case of predicting negative affect after social exclusion, a higher BR decrease during the first block of the game could be linked to a more relaxed state and positive expectancies, while the others’ opinion was still unknown, which could make them more prone to frustration later. On the other hand, a higher BR increase during the completion of the PANAS after the task could be linked to higher anger in the face of social judgement and the labelling thereof. Thus, the combination of both parameters is needed to more accurately determine the negative affect.

These results can give insights to extract relevant features from autonomic physiology that can be considered into monitoring systems. These systems could be aimed to affective computing or biofeedback applications and be easily adapted to ecological settings and social environments. Research studies in which subjects pass both conditions sequentially, in addition to longitudinal studies, would be desirable in order to determine individual trait differences in breathing response to emotional lability.

This study shows some limitations. The comparisons among different conditions used different length epochs, since it was not convenient to limit the time to fill the PANAS. When BR is computed in a whole time segment, the incidence of breath-holding events (e.g., brief apnoea for attentional focus linked to attentional engagement) is not distinguished from slower BR. Moreover, in future research, other features derived from the respiratory signal (e.g., tidal volume) could provide additional information. The sample size was relatively small and only composed of female participants, so the significant differences could have been compromised. Finally, a qualitative analysis of the debriefing with the participants would have been convenient to disentangle the particular weight of the conscious awareness of fewer passes during Cyberball and the weight of feedback from teammates on the feeling of exclusion.

## Data Availability

The data that support the findings of this study are available from the corresponding author upon request.
